# From radial to unidirectional water pumping in zeta-potential modulated Nafion nanostructures

**DOI:** 10.1038/s41467-022-30554-7

**Published:** 2022-05-19

**Authors:** María J. Esplandiu, David Reguera, Daniel Romero-Guzmán, Amparo M. Gallardo-Moreno, Jordi Fraxedas

**Affiliations:** 1 Catalan Institute of Nanoscience and Nanotechnology (ICN2), CSIC and BIST, Campus UAB, Bellaterra, 08193 Barcelona, Spain; 2 Departament de Física de la Matèria Condensada, Universitat de Barcelona, C/Martí i Franquès 1, 08028 Barcelona, Spain; 3 Universitat de Barcelona, Institute of Complex Systems (UBICS), C/Martí i Franquès 1, 08028 Barcelona, Spain; 4 University of Extremadura, Department of Applied Physics and University Institute of Biomedical Research (INUBE), Badajoz, Spain; 5 Networking Research Center on Bioengineering, Biomaterials and Nanomedicine (CIBER-BBN), Badajoz, Spain

**Keywords:** Fluids, Polymers, Fluidics, Surface patterning

## Abstract

Chemically propelled micropumps are promising wireless systems to autonomously drive fluid flows for many applications. However, many of these systems are activated by nocuous chemical fuels, cannot operate at high salt concentrations, or have difficulty for controlling flow directionality. In this work we report on a self-driven polymer micropump fueled by salt which can trigger both radial and unidirectional fluid flows. The micropump is based on the cation-exchanger Nafion, which produces chemical gradients and local electric fields capable to trigger interfacial electroosmotic flows. Unidirectional pumping is predicted by simulations and achieved experimentally by nanostructuring Nafion into microarrays with a fine tune modulation of surrounding surface zeta potentials. Nafion micropumps work in a wide range of salt concentrations, are reusable, and can be fueled by different salt cations. We demonstrate that they work with the common water-contaminant cadmium, using the own capture of this ion as fuel to drive fluid pumping. Thus, this system has potential for efficient and fast water purification strategies for environmental remediation. Unidirectional Nafion pumps also hold promise for effective analyte delivery or preconcentration for (bio)sensing assays.

## Abstract (web summary)

Chemically propelled micropumps are wireless fluid flow driving systems with many potential applications. Here, the authors report a self-driven reusable Nafion micropump fueled by different salt cations in a wide range of concentrations that triggers both radial and unidirectional flows, showing efficient water remediation capabilities.

## Introduction

Interfacial diffusio-osmotic or/and electroosmotic fluid flows triggered by self-generated chemical gradients are the basis behind chemically propelled micro/nanomotors or swimmers. The intense activity in this fascinating field in the last years has provided very appealing demonstrations of multitasking swimmers^1–20^. The immobilized counterparts of micro/nanomotors are micropumps, sharing the same working principle of the swimmers, but driving the flow of the surrounding fluid instead of self-propelling in a fluid at rest^21^. These immobilized versions of motors are also especially suitable for better experimental probing and understanding the phoretic mechanisms behind swimmers^21^. Micropumps are also promising platforms for many applications such as mass release, transport, accumulation, and clearance^5,22^; material patterning at precise locations^23–25^; or in sensing applications^22,26,27^.

Self-powered micropumps of different material composition and working principles have been investigated in recent years. Most studies have focused on catalytic bimetallic or semiconductor/metallic pumps governed by electroosmotic flows^5,21,28–34^. Other type of pumps have combined passive materials with metals, semiconductors, solid salts, polymers or enzymes, switching on ionic, neutral diffusio-osmotic, or density-driven convective flows induced by the gradients of ions or neutral species generated at the active part of the pump^32,35–39^. Fluid pumping has been also achieved by just illuminating a microfluidic chamber with UV light, initiating photochemical reactions in the solution phase which drives flows by solute buoyancy (in the bulk fluid) and diffusion-osmosis (at the walls of the chamber)^27^.

Many of the pumps outlined above are triggered by the hydrogen peroxide (H_2_O_2_) decomposition reaction which can be toxic for some applications, especially in the biological context. Therefore, there is always a need to search for more innocuous chemical fuels or novel reaction mechanisms to overcome such limitations. The use of motors or pumps fed by enzyme substrates is a very elegant way to expand applications in the biomedical field^9,10,37–39^. Another alternative using innocuous fuels are self-powered pumps or swimmers made of ion-exchange polymers. In this context, a pump based on immobilizing particles of ion-exchange resins on glass with the capability of triggering electroosmotic flows with micromolar salt concentrations has been reported^40^. However, most biomedical and environmental remediation applications need to operate at higher salt concentrations.

Another desirable feature of self-powered pumps is to control the directionality of the flow. The vast majority of previous studies have focused on demonstrating fluid pumping through local recirculated flows towards and away from active patches. But achieving fluid flow unidirectionality will expand their applications in the field of drug delivery, biosensing or environmental remediation. An initial strategy of originating directional flows was carried out by Hess and coworkers with a pumping membrane trying to imitate the transport of analytes in biomembranes^41^. In that work a porous membrane coated at both sides with two different noble metals could induce electroosmotic flows through the pores triggered by the decomposition of hydrogen peroxide at the metallic layers when both metal layers were connected by an external switch. Later on, the group of Sen and coworkers came up with a pumping system made of an array of metal/enzymatic catalytic strips that could achieve unidirectional flows by setting-up a gradient of chemical fuel, in this case of H_2_O_2_ with a gel soaked in H_2_O_2_^22^. However, it would be desirable to achieve self-driven unidirectional pumping without the application of external gradients (electrical, chemical, or pressure). Recently the same group has achieved self-organization of fluid flows with a multienzymatic pump system^42^. In a similar context, Fischer and coworkers devised a unidirectional water pumping system using an array of three-dimensional photochemically active Au/TiO_2_ Janus pillars acting as pumping walls^43^. The pillars catalyze the water-splitting reaction under UV illumination, giving rise to local osmotic flows around them. The cooperative effect of the flows generated by an array of pillars leads to unidirectional macroscopic flow for a careful choice of the geometry and arrangement of the pillars.

The scarcity of studies reporting wireless fluid flow unidirectionality without externally imposed gradients might be rooted in the difficulties to match the proper arrangement and pumping mechanism to accomplish a constructive effect using an array of pumps. Fluid flow unidirectionality is not simply achieved by a periodic repetition of active structures. Instead, a suitable geometrical layout and a controlled surface nanoengineering of the active materials are needed to sustain fluid flow motion in one direction.

Here, we report on a self-activated micropump based on Nafion which can pump fluid using salts as chemical fuel and can be nanostructured to achieve unidirectional pumping. Nafion is a perfluorinated sulfonic acid ionomer with high performance cation-exchanger capabilities apart from other attributes such as very high ionic conductivity, excellent thermal and mechanical stability, biocompatibility, and antifouling characteristics^44^. We harness the ion-exchange capabilities of Nafion^44^ to induce both radial or unidirectional tangential electroosmotic flows in the presence of salts^45–48^. To accomplish long-range unidirectional fluid pumping, an array of Nafion microstrips are patterned with highly controllable nanofabrication strategies. These active strips are integrated, in turn, into an array of adjoining strips with different zeta potentials (ζ). Thus, the novelty of this wireless pump system lies in the design of an ion-exchange powering system integrated in a nanofabricated array with fine-tuning modulation of zeta potential surrounding the active Nafion to control the direction of the flow. We prove fluid pumping in a wide range of salt concentrations covering more than four orders of magnitude, from micromolar to the millimolar range. We also show that these pumps can be easily regenerated for reusability without almost no loss of performance. More importantly, we demonstrate that these pumps can be self-driven using different cations as fuel. In particular, we show that these Nafion pumps can work by efficiently capturing heavy metal cations such as cadmium ions present in contaminated water samples. Therefore, these systems might have promising potential applications in water remediation, where one could devise micropump channels in which contaminated water would be self-driven by the own contaminant, delivering purified water at the end of the pumping channel.

This study also expands the versatility of Nafion material from the well-known application areas of the chlor-alkali industry, fuel cell technology^44,49–52^ or biosensor technology^53,54^ to the appealing field of wireless micro/nanofluidic networks and self-propelled micro/nanomotors, promoted by the strategies achieved in Nafion nanopatterning.

## Results and discussion

### Nafion pumps driving radial flows

We first demonstrate the capability of Nafion to trigger fluid pumping in a simple configuration which activates radial flows. The pump layout consists of a 100 µm diameter and about 600 nm thick Nafion disc surrounded by a region in which Nafion has been mostly deactivated. The fresh Nafion material is in its protonated form and deactivation is achieved by electron-beam lithography which scissors Nafion −SO_3_^−^ moieties decreasing its ion-exchange capabilities^47^.

**Fig. 1:**
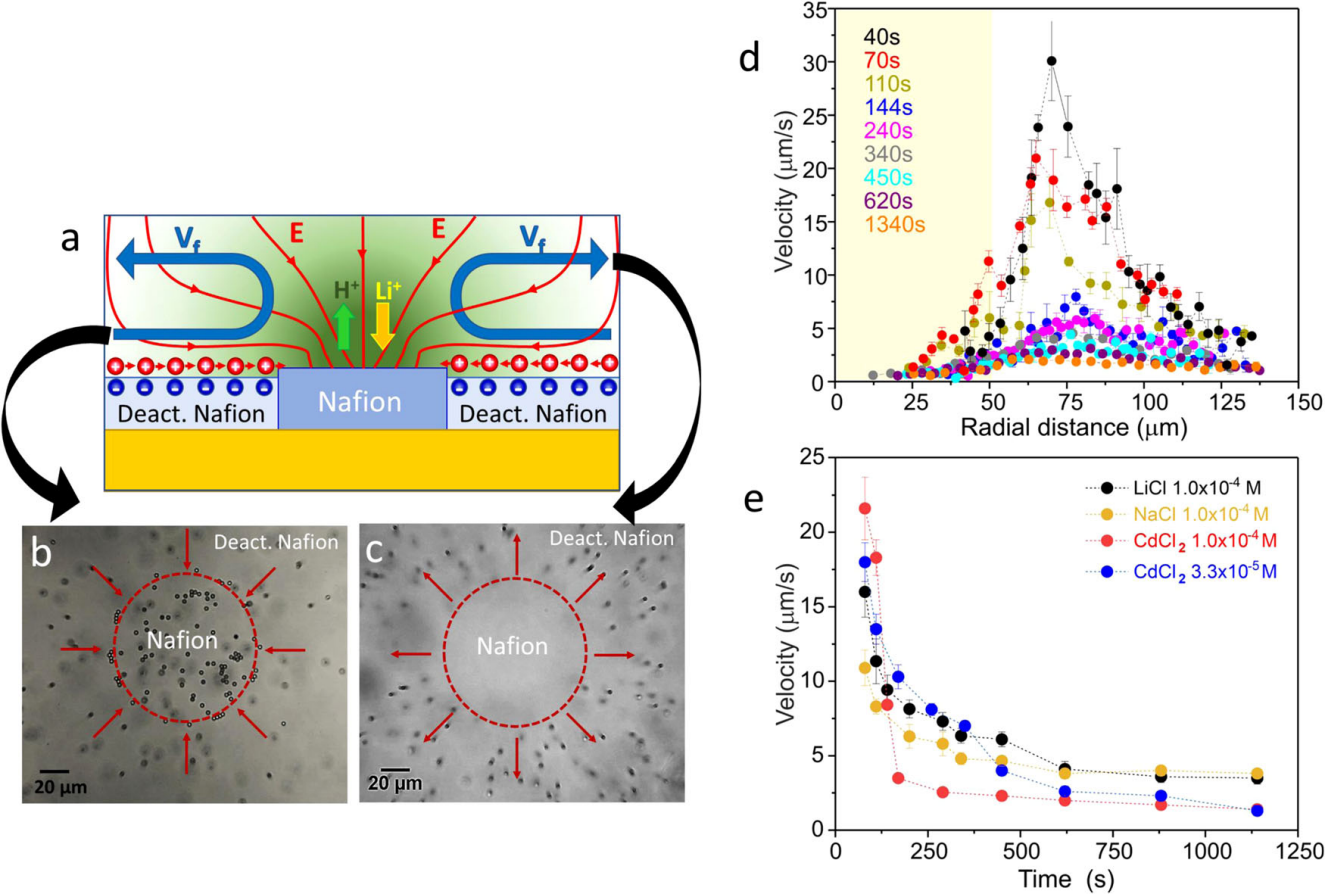
Radial Nafion micropumps. **a** (top) Schematic picture of the cross section of a Nafion micropump, illustrating the pumping mechanism. The ion-exchange generates an ion concentration gradient (in green) and an electric field (**E**) schematized by the red lines, mimicking those obtained from the simulations as described in the Supplementary Figure 3. The charges in the deactivated Nafion illustrate the negative zeta potential of this surface (negative charges at the active Nafion interface are not shown for simplicity). The positive charges in the liquid interface denote the counterions that will be moved by the tangential component of the ion-exchange self-generated electric field, dragging a fluid flow (**V**_f_) towards the active Nafion disc. Near the disc edge, the flow goes up by fluid continuity and finally bends outwards due to the presence of the top wall. **b**, **c** Top view snapshots of the actual Nafion pump taken at different heights above the surface. The polystyrene tracers dragged by the fluid approach the Nafion structure as shown in the image captured at a plane near the surface **b** and then they are lifted by the fluid and move away from the Nafion patch, as shown by the image taken at a *z*-plane located at 100 µm from the surface **c**. **d** Average radial velocity of polystyrene tracers dispersed in 1.0 × 10^−4^ M of LiCl near the surface as a function of the radial distance and at different pumping times. Data of the different curves have been obtained by evaluating and averaging the trajectory of more than 50 polystyrene particles. The yellowish shaded area represents the presence of the Nafion disc from the center of the disc (0 µm) to the edge (50 µm). **e** Time evolution of the maximum radial velocity of tracers averaged over 25–50 trajectories in presence of aqueous solutions containing different salt cations. Error bars in the figures represent the standard deviation. Source data of **d**, **e** are provided as a Source Data file.

Figure 1a schematizes a cross section of the micropump under study depicting the pumping mechanism. Fluid pumping is triggered when the patterned Nafion system is immersed in aqueous salt solutions due to the exchange of protons by the salt cation, in this case exemplified with LiCl aqueous solutions. In the experimental device, the fluid flow is followed by tracking the motion of polystyrene-based tracer particles. Figure 1b and c show the typical motion behavior of the colloidal tracers close (b) and far away (c) from the surface, respectively. Particles close to the surface move toward the Nafion disc, however, as they approach the disc edge their trajectory bends upward in the direction perpendicular to the disc and finally bend outwards the disc due to fluid continuity and the presence of the top wall of the cell (See Supplementary Movie 1 and 2).

The spatial velocity and time evolution of the pump performance was evaluated by tracking the average radial velocities of the polystyrene tracers dispersed in 1.0 × 10^−4 ^M of LiCl at the region close to the surface (Fig. 1b). In general, particles increase their radial velocity when approaching to the disc, exhibiting maximum values at 60–80 µm from the disc center (radius of the Nafion disc is 50 µm). Then the radial velocity decays inside the Nafion region because the particles are pushed up by the fluid or because they are retained on the disc when the speed of the fluid is not large enough to lift them, a phenomenon that is more probable to occur at large times. Maximum tracer velocities of around 30 µm/s have been detected at very short times. These are large values as compared to those obtained with other catalytic pumps combining metals in H_2_O_2_ solutions^30^ or metals/semiconductors in water^31,32^. The maximum velocity was decreasing to values around 2 µm/s when reaching half an hour of actuation. The pump was able to continue running for more than 45 minutes, which is remarkable considering that the layer of Nafion is only 600 nm thick.

Nafion pumps can be activated with different salts as fuel since Nafion can exchange protons with different cations^47^ (Supplementary Movies 3 and 4). In fact, Fig. 1 e shows comparatively the time evolution of the pumping velocity triggered with LiCl, NaCl, and CdCl_2_ of same concentration (1.0 × 10^−4^ M) and with CdCl_2_ of the same ionic strength than the alkali metal chlorides (CdCl_2_ 3.3 × 10^−5^M). Very high velocities have been observed at shorter times being those obtained with the divalent cations the higher ones and also those that decay more abruptly with time, especially the one at 1.0 × 10^−4^ M of CdCl_2_.

The effect of salt concentration on the pump performance has also been evaluated experimentally as a function of different concentrations of LiCl as depicted in Supplementary Fig. 2a. The radial fluid velocity of the tracers exhibits a non-monotonic behavior; it increases with salt concentration at very low concentrations of salts but then starts decreasing at higher concentrations. Fluid pumping remarkably persists even at mM salt concentrations. Thus, these Nafion pumps are very promising for applications since they can be operative in a wide range of salt concentrations, in contrast with catalytic pumps.

Another very interesting feature of these pumps is their reusability after regeneration. The fresh Nafion pump is in its protonated form and after use with LiCl electrolytes the pump is loaded with Li^+^ ions. The Nafion pump was regenerated to the protonated form by immersion in 10^−2^ M HCl for 6 h. Then the pump was reused in 1.0 × 10^−4^ M LiCl. Supplementary Figure 2b compares the radial velocity of tracers in 1.0 × 10^−4^ M LiCl on a freshly prepared pump with that obtained when using the same pump for a second time after a regeneration step. The pump performance was almost the same, with only a slight decrease in speed after regeneration.

### Ion-exchange driven fluid pumping

The mechanism responsible for fluid pumping is illustrated schematically in Fig. 1a. Pumping originates from the Nafion capability to exchange ions. When a protonated Nafion sample is immersed in a salt-containing electrolyte, such as LiCl, protons are released from Nafion and in exchange, lithium ions from the electrolyte are incorporated into the Nafion film. As a consequence, gradients in the concentration of protons and lithium ions built-up near the interface^47^. These gradients will generate an ion current governed by the Nernst-Planck equation

1 $${{{{{{\bf{j}}}}}}}_{{{{{{\rm{i}}}}}}}=-{D}_{i}\nabla {C}_{i}+\frac{{z}_{i}e{D}_{i}}{{k}_{B}T}{C}_{i}{{{{{\bf{E}}}}}}+{{{{{\bf{v}}}}}}{C}_{i},$$

where the terms on the right-hand side represent the diffusion (due to concentration gradient), migration (due to an electric field **E**), and convection (due to a fluid velocity **v**) contributions, respectively. *D*_*i*_ and *C*_*i*_ stand for the diffusion coefficient and concentration of the ion *i*, *z*_*i*_ is the ion valence, *e* is the elementary charge, ${k}_{B}$ is Boltzmann’s constant, and *T* is the temperature. This ion exchange does not generate a net *electric* current ${{{{{{\bf{J}}}}}}}_{{{{{{\bf{e}}}}}}}$. Therefore, by imposing $\it {{{{{{\bf{J}}}}}}}_{{{{{{\rm{e}}}}}}}=\mathop{\sum}\limits_{i}{z}_{i}e{{{{{{\bf{j}}}}}}}_{{{{{{\rm{i}}}}}}}$ = 0, one obtains that an electric field

2 $$\it \it {{{{{\bf{E}}}}}}=\frac{{k}_{B}T}{e}\frac{\mathop{\sum}\limits_{i}{z}_{i}{D}_{i}\nabla {C}_{i}}{\mathop{\sum}\limits_{i}{z}_{i}^{2}{D}_{i}{C}_{i}}$$

builds up in the presence of concentration gradients in order to guarantee that there is no electric current. The generation of an electric field of similar origin is the basic principle behind ionic diffusiophoresis, upon which many catalytic motors and pumps operate. (The convective term is usually negligible and actually does not contribute to the net charge current if the fluid is electroneutral. Electroneutrality also yields a zero electric field if the diffusion coefficient of the ions ${D}_{i}$ are all the same). Hence, a concentration gradient and an electric field are spontaneously built-up near the interface due to the unequal diffusion coefficients of the exchanged ions as proved by previous studies^47,55^. Among the salts containing alkali metal chlorides, Li ions were chosen for the experiments due to their low diffusion coefficient, thus providing higher difference of diffusion coefficients with respect to protons, which results in larger electric field generation near the interface.

When a continuum layer of Nafion is used, the concentration gradients and the electric field are perpendicular to the surface, and do not generate any fluid flow. For triggering fluid motion a tangential component of the electric field is needed. This is achieved by nanostructuring Nafion into patches, as the one depicted in Fig. 1a, representing a Nafion disc surrounded by deactivated Nafion. For this finite Nafion patch the electric field will still point towards the Nafion, but it will now have a component parallel to the surface that, when this surface is charged, will lead to fluid flow by multi-ion diffusiophoresis^47,55^. In a multi-ion solution the diffusio-osmotic velocity of the fluid is given by^55^:

3 $${{{{{{\boldsymbol{v}}}}}}}_{{{{{{\boldsymbol{do}}}}}}}=-\frac{\varepsilon }{8\eta }\frac{{\sum }_{i}{z}_{i}^{2}\nabla {C}_{i}}{{\sum }_{i}{z}_{i}^{2}{C}_{i}}{\zeta }_{w}^{2}-\frac{\varepsilon }{\eta }{{{{{\bf{E}}}}}}{\zeta }_{w},$$

where the first term represents the chemi-osmotic contribution and the second one the electroosmotic term that is proportional to the electric field given by Eq. (2), being *ζ*_*w*_ the surface zeta potential and $\varepsilon$ and $\eta$ the permittivity and the viscosity of the fluid, respectively. We found that the electroosmotic contribution is the dominant term^47^. Accordingly, the direction of the fluid flow is dictated by the sign of the surface’s zeta potential^56^. We have measured experimentally the zeta potential of Nafion and deactivated Nafion from the streaming potential /current of planar surfaces of these materials using 1.0 × 10^−4 ^M LiCl as electrolyte and pH of 5.7. The Nafion region is the one with largest negative zeta potential (−73 ± 3 mV). The deactivated Nafion exhibits less negative zeta potential (−37 ± 3 mV) with respect to Nafion which is expected due to the removal of sulfonic groups. Since the zeta potential of the surface (both deactivated Nafion and Nafion) is negative, tangential electroosmotic and chemi-osmotic fluid flows towards the Nafion disc are switched on with the consequent formation of convection rolls.

This mechanism has been verified by simulations mimicking the experimental setup. The details of the implementation are explained in the Supplementary methods. Supplementary Figure 3 shows the proton concentration, the electric field, and the fluid flow streamlines resulting from the simulation of the radial Nafion pump. The simulation shows that the exchange of protons by Li^+^ ions taking place at the Nafion disc generates a concentration gradient and an electric field pointing radially towards the Nafion disc. The tangential component of this electric field acting on the positive mobile counterions accumulated on the negatively charged deactivated Nafion surface, drags the fluid towards the Nafion disc generating convection rolls. Supplementary Figure 4 shows the value of the radial component of the electric field as a function of the radial distance, measured from the center of the Nafion disc, depicting a strong peak at the disc edge. The simulations also show that this tangential component of the electric field is almost insensitive to the value of the zeta potential of the material surrounding the active Nafion. The zeta potential of the surface sets the sign of the charge and distribution of mobile counterions that move in the presence of the tangential electric field, dragging the fluid along. Simulations also confirm that there is no fluid flow in the absence of ion-exchange, and that there is pumping even if the zeta potentials of both surfaces would be the same.

Supplementary Figure 5 shows the behavior obtained in the simulation for the pumping velocity as a function of salt concentration. If Nafion acts as a pure sink for cations, the electric field and the pumping velocity are expected to increase with salt concentration up to reaching a constant value at high salt concentrations (see Supplementary Fig. 5a and b). In fact, Eq. (2) helps explaining this behavior and the fact that these pumps can still work at high salt concentrations, unlike catalytic pumps. In catalytic pumps, the current and the self-generated electric field are dictated by the fuel concentration, typically hydrogen peroxide. The addition of salt increases the ionic strength and screens the electric field, thus reducing the driving force and making this kind of motors and pumps almost inoperative at high salt concentrations. Contrarily, for ion-exchange pumps, since the salt itself is the fuel, increasing the salt concentration will increase the concentration gradient (the numerator in Eq. (2)) and the ionic strength (the denominator) in a similar way, leading to an electric field and a corresponding velocity flow which stay almost constant for high salt concentrations, as observed in the simulations.

The decay of the velocity for high salt concentrations (Supplementary Fig. 2a) as well as the decline in performance in time observed in the experiments (Fig. 1 e) is due to the limited proton capacity of the thin Nafion layer used. A simple order of magnitude estimate can be done to illustrate this effect. The ion exchange capacity of Nafion is about 4 × 10^3^ mols of H^+^/m^3^ ^47^, which for a 0.6 µm thick Nafion film yields 2.4 × 10^−3^ mols of H^+^/m^2^. The average diffusion current of the Li^+^ cation at the Nafion interface obtained in the simulations is 3.8 × 10^−6 ^mol/m^2^ s. That means that after roughly 630 s, all the protons will be exhausted if the exchange proceeds at the steady state rate. Presumably, as protons get scarced the exchange current will get smaller, reducing the electric field and the pump velocity. Since the exchange current is proportional to the salt concentration, this saturation process will occur faster for higher salt concentrations. Accordingly, due to a faster saturation, the pumping speed will vanish quicker and at a fixed time, the velocity is expected to be smaller as the salt concentration gets higher.

Simulations (see Supplementary Fig. 6) also show that the pumping works with similar velocities with different monovalent cations as Li^+^, Na^+^, K^+^ and also with divalent ions as Cd^+2^. In the case of the divalent ion, 2H^+^ are exchanged by one Cd^2+^. Therefore, saturation would be reached at shorter times, which explains the rapid drop in pump speed observed in the experiments with Cd^+2^.

Supplementary Figure 8 shows the experimental verification that large pumping speeds can be sustained longer times by increasing the thickness of the Nafion layer, since its saturation is delayed. Thus, we believe these polymeric pumps could have the capability to operate at concentrations of salts above the millimolar range by tuning the thickness of the polymeric layer. Future work will be done to address specifically this issue, and the details of the ion-exchange mechanism that go beyond the scope of the present paper, where the goal was to demonstrate the feasibility to use ion-exchange to generate fluid pumping and achieve unidirectional flow.

### Nafion pump driving unidirectional flows

**Fig. 2:**
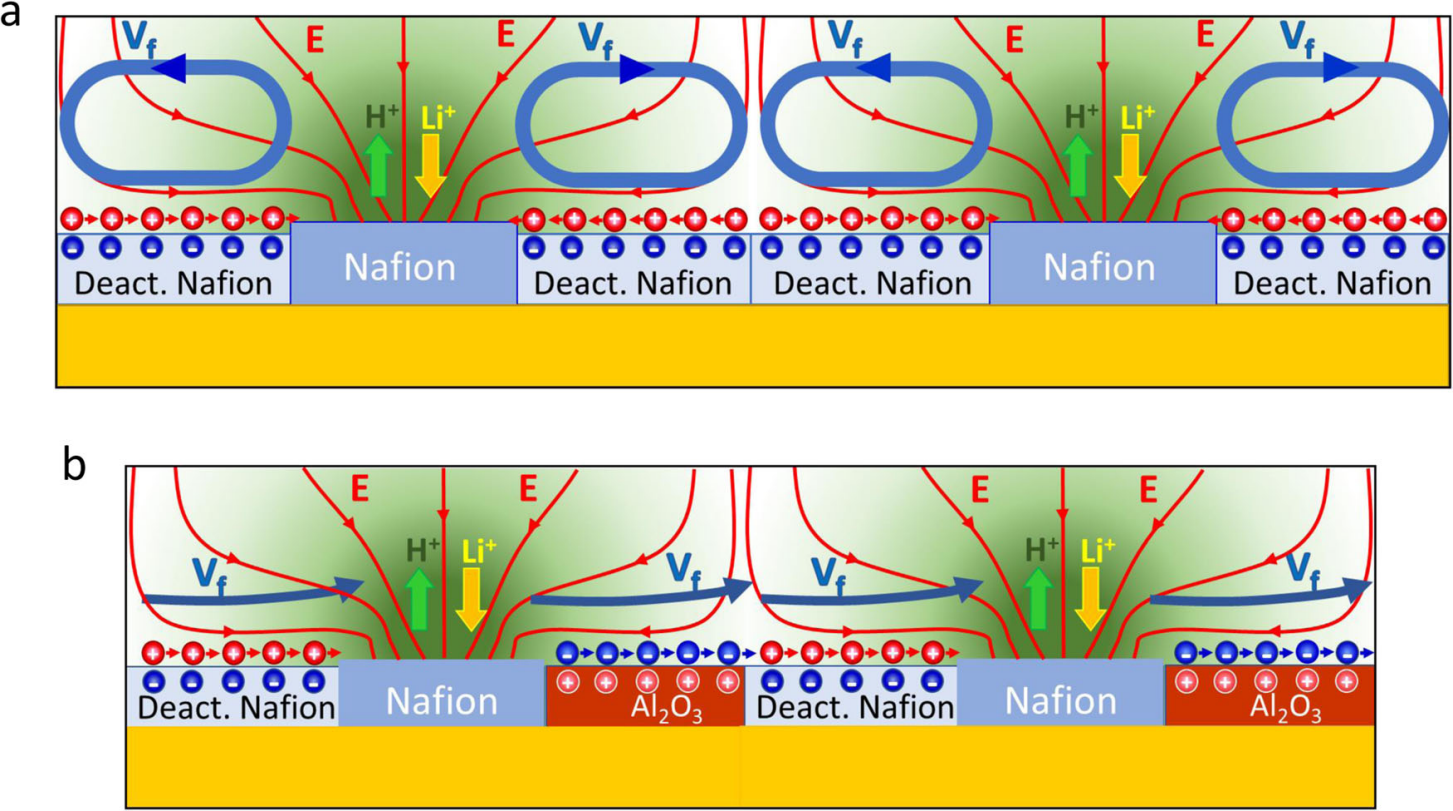
From convective rolls to unidirectional pumping. **a** Scheme of a pump made of alternating strips of deactivated and active Nafion, leading to the formation of multiple convection rolls along the patterned array. **b** Scheme of the design of a pump based on the periodic repetition of a basic unit made of alternating strips of deactivated Nafion (negative ζ)/Nafion/Al_2_O_3_ (positive ζ) which would lead to unidirectional fluid flow along the patterned surface. The charged interface in the Nafion has been omitted to lighten the content of the figure. The Al_2_O_3_ patches with positive ζ accumulate negative counterions that in the presence of the tangential component of the electric field generated by the ion-exchange will move also to the right, dragging the fluid along to the next repeating unit, achieving unidirectional flow.

The most common strategy to sustain unidirectional fluid pumping over long distances is to repeat periodically the basic structure that generates fluid motion. However, in this case the combination of alternating strips of active and deactivated Nafion would not accomplish unidirectional flow since multiple convection rolls would be produced instead. The electric field pointing towards the active Nafion pad together with the symmetric surface zeta potential at both sides would always direct the fluid flow towards the active Nafion patches as illustrated in Fig. 2a. To rectify the fluid motion in one direction it is important to break the surface charge symmetry around Nafion. A simple way to accomplish that is by adding a third stripe of a material with positive ζ potential. The electric field generated by the ion-exchange in the Nafion pad acting on the region of positive zeta potential would drive an electroosmotic flow now pointing outwards from the Nafion patch, redirecting the fluid flow to the following pump unit of the array as illustrated in Fig. 2b. Therefore, by periodically repeating a basic structure formed by alternating strips of deactivated Nafion (negative ζ) / Nafion / surface with positive ζ, the convective rolls could be suppressed, achieving the desired unidirectional pumping.

**Fig. 3:**
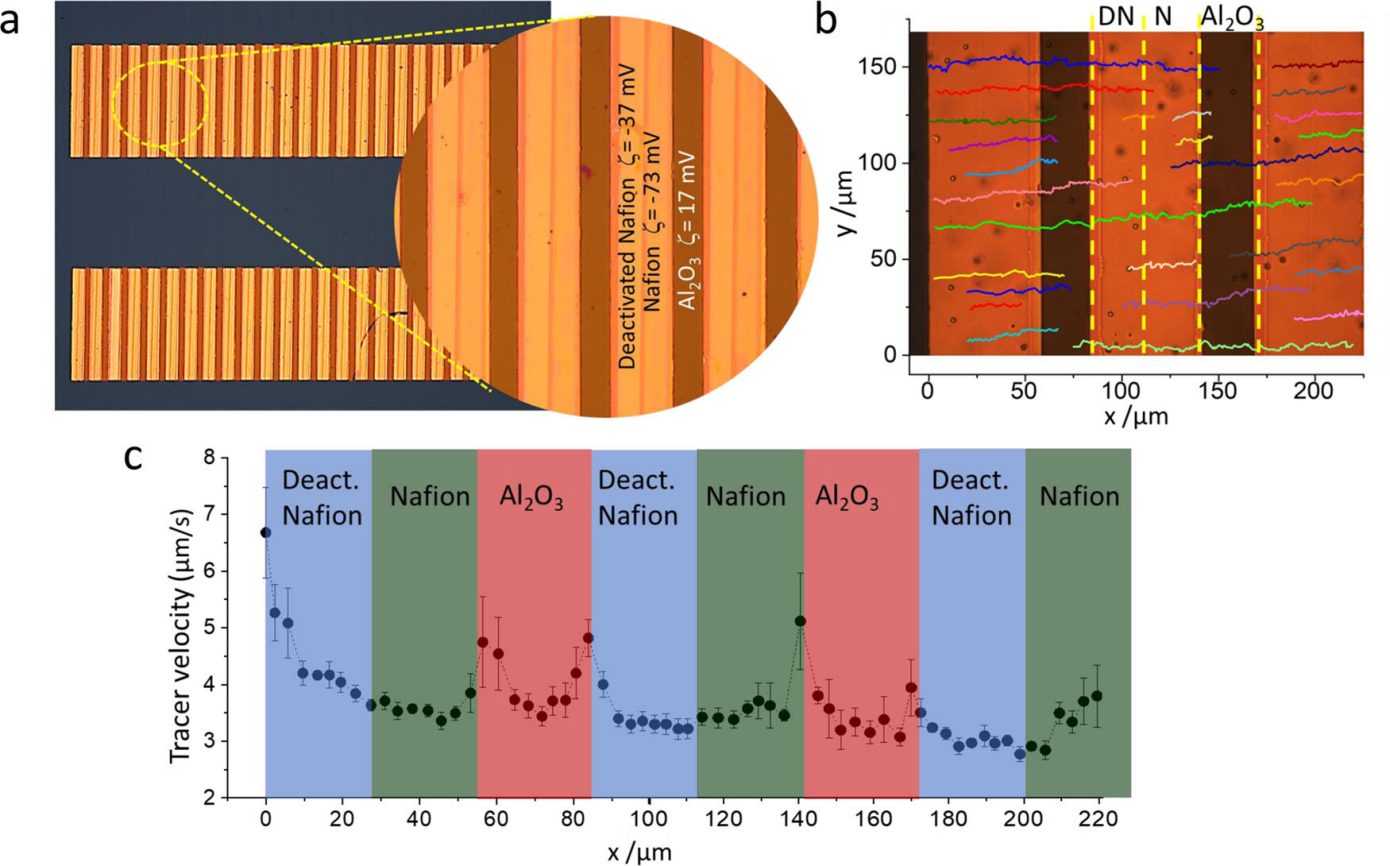
Unidirectional pumping with deactivated Nafion/Nafion/Al_2_O_3_ nanostructures. **a** Patterned array of strips made from the repetitive unit composed of deactivated Nafion (25 μm, ζ = −37 ± 3 mV) /Nafion (25 μm, ζ = −73 ± 3 mV) /Al_2_O_3_ (30 μm, ζ = 17 ± 2 mV). **b** Tracer trajectories along the patterned surface proving directional motion (DN and N refer to deactivated Nafion and Nafion, respectively). **c** Average tangential velocity of polystyrene tracers along the patterned structure. Blue, green and red shaded areas represent deactivated Nafion, Nafion and Al_2_O_3_ strips, respectively. Error bars in the figure represent the standard deviation. Source data of **b**, **c** are provided as a Source Data file.

Accordingly, we have fabricated micropump units consisting of three adjacent strips made of deactivated Nafion, Nafion, and Al_2_O_3_ with 25, 25, and 30 µm width, respectively, and 500 µm long (Fig. 3a). These elements constitute the repetitive unit in a patterned array extended in space. The self-pumping system was patterned using standard electron-beam lithography, electron-beam evaporation, and spin coating as detailed in Methods and Experimental Section. The process is also compatible with photolithography or the use of stencil lithography. The zeta potential of alumina was also measured at 1.0 × 10^−4 ^M LiCl and pH of 5.7, verifying that it has a positive value of 17 ± 2 mV. As before, the fluid motion is followed by tracking the motion of polystyrene particle tracers in 1.0 × 10^−4^ M LiCl.

Figure 3b shows a bunch of trajectories of the polystyrene tracers when interacting with the patterned pump, proving their unidirectional motion. The average velocity of tracers along the patterned surface during the first ten minutes of actuation is depicted in Fig. 3c. In general, the tracers move with average velocities above 3 µm/s, which can be speed up at the boundaries of the different materials. The motion of particle tracers can be observed in Supplementary Movie 5.

**Fig. 4:**
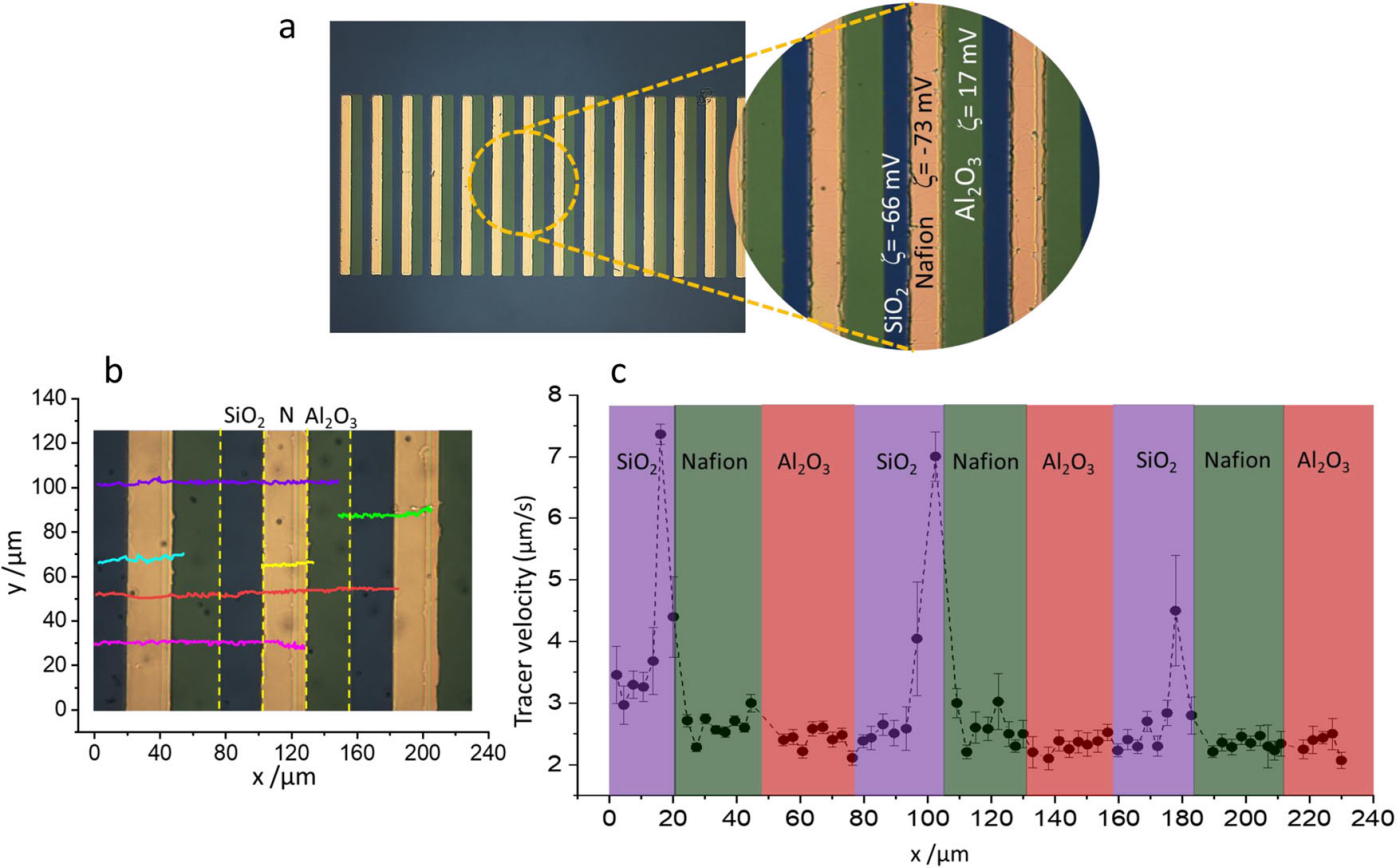
Unidirectional pumping with SiO_2_/Nafion/Al_2_O_3_ nanostructures. **a** Patterned array of strips made from the repetitive unit composed of SiO_2_ (25 μm, ζ = −66 ± 3 mV) /Nafion (25 μm, ζ = −73 ± 3 mV) /Al_2_O_3_ (30 μm, ζ = 17 ± 2 mV). **b** Tracer trajectories along the patterned surface proving directional motion (N refers to Nafion). Source data are provided as a Source Data file. **c** Average tangential velocity of tracers along the patterned structures. Polystyrene tracers in 1.0 × 10^−4^ M LiCl have been used to track the fluid flow. Violet, green and red shaded areas represent SiO_2_, Nafion, and Al_2_O_3_ strips, respectively. Error bars in the figure represent the standard deviation. Source data of **b**, **c** are provided as a Source Data file.

We have also designed another pump array by replacing the deactivated Nafion with SiO_2_ structures. Silicon oxide exhibits a more negative zeta potential than the deactivated Nafion structure with a value of −66 ± 3 mV. The repetitive pumping unit consists in this case of alternating strips of SiO_2_/Nafion/Al_2_O_3_ with dimensions of 25, 25, and 30 µm width, respectively, and 500 µm long (see Fig. 4a). Unidirectional fluid flow has been accomplished as can be proved from the tracer trajectories collected in Fig. 4b and Supplementary Movie 6. We have extracted the average velocity along the patterned array by evaluating the motion of the tracers during the first ten minutes of the pump actuation as depicted in Fig. 4c. Similar pump performance has been achieved as compared to the deactivated Nafion/Nafion/Al_2_O_3_ with average velocities above 2 µm/s together with velocity peaks mainly at the SiO_2_/Nafion boundaries. Control experiments in absence of salt show only Brownian motion in the tracer particles (Supplementary Movie 7).

Another cross-shaped pump configuration was fabricated using four arrays of these strips made from repetitive units of SiO_2_/Nafion/Al_2_O_3_ as depicted in Supplementary Fig. 9. This layout serves to demonstrate that the fluid can be redirected simultaneously in different orthogonal directions, as can be seen in the Supplementary Movie 8.

We have also contrasted these experimental data with finite element simulations. The implementation details are discussed in the Supplementary Information. A micropump array of 5 repeating units, each of them composed of alternating strips of SiO_2_/Nafion /Al_2_O_3_ with the same stripe dimensions and zeta potentials than in the experiment, was simulated. The rest of parameters are listed in Supplementary Table 1.

**Fig. 5:**
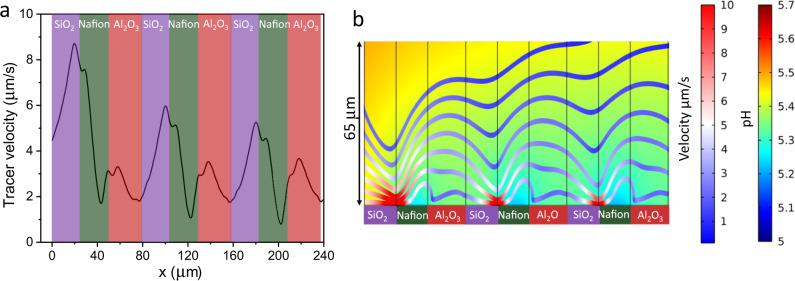
Simulations of unidirectional micropumps. **a** Horizontal component of the expected velocity of a polystyrene tracer particle along the strip array composed by repetitive micropumps of SiO_2_/Nafion/Al_2_O_3_. The tracer velocity was obtained from the simulations at 3 µm above the surface and was smoothed using a 5 µm moving average. Source data are provided as a Source Data file. **b** pH mapping (color scale) and streamlines of the fluid flow over the first three repetitions of the periodic structure. The streamlines are colored by the local value of the fluid velocity, and illustrate the variations in speed caused by the local pumping triggered by the Nafion patches.

Figure 5a shows the value of the horizontal component of the velocity of a polystyrene tracer particle obtained from the simulations at 3 µm above the surface. The simulations reveal the net motion of the tracer along the micropump array with average velocities above 2 µm/s and support the generation of a directional fluid flow. The tangential velocity exhibits periodic variations with large peaks at the SiO_2_/Nafion boundaries and small peaks at the Nafion/Al_2_O_3_ interface. This simulation results agree qualitatively with the experimental data shown in Fig. 4c. A direct, quantitative comparison is hard to perform since the tracer particles do not follow a trajectory of constant height due to the influence of gravity, Brownian motion, and the vertical component of the velocity. The velocity peaks coincide with the regions of higher chemical gradients, as can be observed in the pH mapping of Fig. 5b. The pH map depicts an increase of proton concentration just above Nafion due to the ion-exchange building up a strong chemical gradient at the neighboring strips. This gradient generates an electric field (see Supplementary Fig. 7) with a tangential component which is positive (i.e., pointing to the right) at the SiO_2_/Nafion boundary and negative (i.e., pointing to the left) at the Nafion/Al_2_O_3_ border. This positive tangential electric field acting above the negatively charged SiO_2_ and negative electric field acting above the positive Al_2_O_3_ surface, both drive a fluid flow in the same direction (towards the right).

The streamlines of the resulting fluid velocity field are also depicted in Fig. 5b, colored by the modulus of the velocity. The fluid velocity is large and shows strong intensity variations near the surface as can be observed from the changes in color along the streamlines. The flow becomes slower and more uniform along the array at higher z distances, with average velocities still exceeding 1 µm/s at 50 µm above the surface.

### Potential application of Nafion pumps for water remediation

As a proof of concept, we have tested experimentally the capabilities of these Nafion micropumps to scavenge Cd^2+^ from water samples. Nafion samples of 2 × 2 cm^2^ and 600 nm thickness were immersed in 3 ml of different ion-containing aqueous solution for 45 min. The initial and final ion concentrations after Nafion interaction were analyzed through optical emission spectrometry with an inductively coupled plasma excitation source (ICP-OES). Table 1 shows the changes in ion concentration of different aqueous samples containing only Cd^2+^ (a) or a mixture of Cd^2+^ and Na^+^ (b and c). In the case of sample (c) the concentration of Na is the typical one found in drinking water.

**Table 1:** Changes of ion concentration of different aqueous solutions containing sodium and cadmium cations after interacting with Nafion devices.

Sample	Initial concentration (C_o_)	Final concentration (C_f_)	Removal percentage
(a) CdCl_2_	11.0 mg/L	0.3 mg/L	97%
(b) CdCl_2_+NaCl	[Cd^2+^] = 11.0 mg/L	[Cd^2+^] = 0.2 mg/L	98%
	[Na^+^] = 2.3 mg/L	[Na^+^] = 2.0 mg/L	13%
(c) CdCl_2_+NaCl	[Cd^2+^] = 11.0 mg/L	[Cd^2+^] = 0.4 mg/L	96%
	[Na^+^] = 23.0 mg/L	[Na^+^] = 21.0 mg/L	9%

Surprisingly, the Nafion pump is quite selective to the removal of cadmium ions and exhibits a very good removal efficiency of more than 95% in very short times of operation with a capacity removal of more than 78 mg of Cd ions per gr of Nafion, equivalent to 1200 moles of Cd^2+^/m^3^ and close to the nominal Nafion ion-exchange capacity. The selectivity of Nafion to Cd^2+^ ions, its reusability, and the potential to work at high salt concentrations, encourage further investigations to optimize the design of these unidirectional self-driven pumps for water decontamination applications. These studies can be expanded to other multivalent ions of interest in biomedicine or environmental remediation.

In summary, we have introduced a chemically powered micropump based on Nafion which is self-activated by its ion-exchange capabilities using innocuous salts as fuel. The exchange of ions with different diffusion coefficients generates an electric field which, by properly arranging the Nafion into patches, can drive fluid flow by electroosmosis. We have demonstrated high-speed fluid pumping forming convection rolls but also unidirectional pumping upon adequate micro/nanostructuring of Nafion by modulating the zeta potential of the adjacent strips. Unidirectional flow is achieved with a micropump array patterned with repetitive units consisting of alternating strips of materials with negative zeta potential (such as deactivated Nafion or SiO_2_), Nafion, and materials with positive zeta potential (e.g., Al_2_O_3_). The interplay of the tangential component of the electric field, generated by the ion-exchange and pointing always towards Nafion, with the fine tuning of the zeta potential of the surrounding strips redirects the fluid flow to the next micropump unit of the array, achieving the desired unidirectional flow. Numerical simulations of these pumps also support the experimental findings. As proof-of-concept application, we have used these Nafion pumps to scavenge selectively and efficiently cadmium ions from water samples with the particularity that the pumping is self-driven by the own contaminant ion.

With this study, we have shown the possibility of driving and guiding self-sustained fluid flows at the microscale by combining ion-exchange with a proper micropatterning of surfaces with adequate zeta potentials. In contrast to other chemically propelled pumps, ion-exchange pumps present high salt tolerance, rooted in the fact that the screening of the electric field caused by the increased ionic strength is also balanced by the increase of the electric field generated by salt concentration gradient. In particular, we have found that these Nafion micropumps can be operative in a wide range of salt concentrations covering more than four orders of magnitude, a remarkable result considering the thin Nafion layer used in this study. Moreover, they can be easily regenerated for reusability. All these properties can become very relevant for optimizing many applications such as mass transport and material patterning at precise locations, (bio)sensing, drug delivery or, environmental applications.

Our results encourage further work on these systems since there is still a lot of room for device optimization. By adjusting Nafion dimensions, it is possible to increase the operation time and to extend its applicability to fluids with higher saline concentrations. Other lithography techniques could be used such as stencil lithography or lithography based on high power lasers with cutting capacity. These are faster fabrication tools and would leave less residues which could increase fluid pumping velocities. Moreover, cost effective plastic materials can be also used as Nafion support which have already shown good adherence and versatility for patterning. The integration of Nafion with adjacent metal pads could be used to change the surrounding zeta potential at will in real time via external stimuli, so that pumping can be turned on and off, or even switched between different directions. All these ingredients are very promising to devise prototypes with practicality and scalability for real applications.

Moreover, this study holds promise to expand the versatility of Nafion material from well-known application areas (e.g., fuel cells, biosensors, filtration/separation/purification technologies, novel antifouling coatings, etc.) to the appealing field of wireless micro/nanofluidic networks or self-propelled micro/nanomotors. In addition, the strategies achieved in Nafion nanopatterning open the possibility to integrate Nafion with metals, insulators, and semiconductors in nanofabricated devices. All these capabilities can pave the way to exploit the remarkable properties of this ion-exchange polymer in myriads of novel applications.

## Methods

### Micropump fabrication

1. **Nafion micropump for triggering radial flows**
   The fabrication of Nafion micropumps started by spinning a diluted Nafion dispersion (5%) in isopropanol from a commercial Nafion dispersion in the protonated form (Aldrich, 10% in water, eq. weight 1100) on Si wafers modified with a 50 nm Au layer and followed by a heating step at 100 °C for 5 min. The spinning process was repeated to achieve typical thicknesses of about 600 nm as measured with an Atomic Force Microscope and a profilometer. After that, the Nafion film was subjected to electron-beam lithography to define a Nafion disc of 100 µm of diameter. The e-beam lithography modifies Nafion composition by the scission of −SO_3_^−^ moieties which yield to a large loss of its ion-exchange capabilities^47^.
2. **Nafion micropump for triggering unidirectional flows**

The fabrication process starts by patterning a gold strip array on a SiO_2_ wafer with strip dimensions of 50 nm thick, 50 µm wide and 500 µm long, and a pitch of 80 µm. The patterning is made by standard electron-beam lithography plus gold evaporation. Then the surface was subjected to an oxygen plasma process (400 W, 2 minutes) followed by the spin coating of a 600 nm thick Nafion layer on the whole surface as described above. Taking advantage of the good adhesion of Nafion on Au, the sample was then immersed in water which removes the Nafion from the silicon parts remaining the Nafion layer only on the gold strips. A second lithographic step was performed to define an array of adjacent Al_2_O_3_ strips. The alumina strip was evaporated with an electron-beam source and had dimensions of 30 µm wide, 500 µm long, and 50 nm thick with a pitch of 80 µm. When doing this second lithographic step with the sample already containing Nafion, the development was done in methylisobutyl ketone without being diluted in isopropanol and for the lift-off dichloromethane was used instead of acetone to avoid Nafion degradation. After that, an area of 25 µm wide x 500 µm long of the gold/Nafion strip was subjected to an electron-beam lithography to deactivate Nafion and thus obtain the pump unit consisting of alternating strips of deactivated Nafion/Nafion/Al_2_O_3_.

In the case of the SiO_2_/Nafion/Al_2_O_3_ micropump array, the fabrication process consisted of two lithographic steps. The first one was to define the Au strip patterns but in this case with a width of 25 µm and keeping the rest of the dimensions the same. The same procedure as mentioned above was followed for defining the Nafion regions on the Au strips and the alumina strips.

### Zeta potential measurements of large surfaces

The zeta potential of Nafion, deactivated Nafion, Al_2_O_3_, and SiO_2_ surfaces was obtained from streaming current measurement using an Electrokinetic Analyzer (EKA, Anton Paar KG, Graz, Austria). The samples were prepared on 4 × 5 cm and 0.2 cm thick polycarbonate plates on which Nafion also exhibit a very good adhesion and is also a compatible substrate for Nafion nanostructuration with electron-beam lithography. In all cases, a commercial clamping cell (Anton Paar) has been used with a PMMA sample (given by the manufacturer) facing the sample to be measured. The system was operated in an alternating pressure ramp form from 0 to 300 mbar. Each measurement was the average of 8 cycles using a 10^−4 ^M LiCl electrolyte solution in Millipore water.

The zeta potential has been calculated using the Helmholtz-Smoluchowski equation:

4 $${\zeta }_{{cell}}=\frac{\eta }{\varepsilon }\frac{L}{S}\frac{d{I}_{{str}}}{{dp}},$$

where ε and η are the permittivity and the viscosity of the electrolyte, respectively; *L* and *S* are the length and the cross section of the electrokinetic channel and *p* and *I*_str_ are the pressure and the streaming current difference between both ends of the channel, respectively. The value of L/S is the same for all measurements made and has been calculated in a previous work^57^: *L*/*S* = 24.5 ± 0.5 mm^−1^. Specifically, this value was obtained experimentally by measuring the seven channels of the PMMA sample provided by the manufacturer, using an optical profilometer (Leica DCM8) for the section measurements, with a resolution of 12 µm in the *Z*-axis and 2.58 µm in the XY-axis, and a micrometer (Schut electronic micrometer) for the length measurements, with an accuracy of 1 µm. The theoretical values provided by the manufacturer were: *L* = 20 mm, *S* = (1 × 0.14) × 7 mm^2^.

Due to the asymmetry of the cell used, the following equation has been applied to obtain the zeta potential of each sample:

5 $${\zeta }_{{cell}}=\frac{1}{2}{\zeta }_{{PMMA}}+\frac{1}{2}{\zeta }_{{sample}}\to {\zeta }_{{sample}}=2{\zeta }_{{cel}l}-{\zeta }_{{PMMA}},$$

where ζ_PMMA_ has been calculated with Eq. 4 using the reference PMMA sample, and its value is: −54 ± 3 mV.

### Fluid flow tracking

The fluid cell was set-up by placing a gasket-like spacer of 8 mm diameter and 0.3 mm thick on top of the pumps and then capped with a thin glass cover. The fluid flow was followed by tracking the motion of 2 µm diameter polystyrene tracer particles (ζ = −12 mV from Kisker Biotech GmbH & Co). Amidine-modified polystyrene particles (ζ = 46 mV, Invitrogen) were also used to obtain Fig. 1. The particle ζ values were obtained with a Malvern ZetaSizer. The colloidal particles were dispersed in different concentrations of LiCl salts ranging from 1.2x 10^−6^ M to 0.001 M. LiCl salts were chosen since previous work demonstrated that a higher electric field was generated at the Nafion interface when protons are exchanged by Li^+^ ions, due to the higher difference of diffusion coefficient values between protons and Li^+^. Before the evaluation of the fluid pumping with colloidal tracers dispersed in LiCl salts, the pump devices were pre-moistened in Milli-Q water for 30 min. Nafion pre-wetting helps to protonate the sulfonate moieties and to minimize the fluid motion due to water uptake when inspecting the fluid pumping. The liquid cell was defined using a silicon spacer (Secure-Seal™, Invitrogen) of 9 mm diameter and capped with a cover glass. The motion of particles was optically recorded and analyzed with Diatrack software to determine their trajectory and velocity.

### Evaluation of Cd^2+^/Na^+^ ion removal

The analysis of the ion concentration before and after interacting with Nafion pumps was carried out by means of optical emission spectrometry with an inductively coupled plasma excitation source (ICP-OES) Agilent, model 5900. The aqueous solutions were previously acidified with HNO_3_ (1% v/v) before being injected, preparing in parallel the corresponding blank (HNO_3_ 1%) and the standard solutions for the calibration process. The concentrations of the standard solutions for both Na^+^ and Cd^2+^ were 0.02, 0.1, 0.25, 1, 2.5, and 5 ppm, all of them in 1% of HNO_3_.

## Supplementary information

Supplementary Information

Description of Additional Supplementary files

Supplementary Movie 1

Supplementary Movie 2

Supplementary Movie 3

Supplementary Movie 4

Supplementary Movie 5

Supplementary Movie 6

Supplementary Movie 7

Supplementary Movie 8

## Data Availability

All data needed to evaluate the conclusions in the paper are present in the paper and/or the Supplementary Information. The source data underlying Figs. 1d, e, 3b, c, 4b, c, 5a, Supplementary Fig. 2a and b, Supplementary Fig. 4, Supplementary Fig. 5a and b, Supplementary Fig. 6a and b, Supplementary Fig. 7, Supplementary Fig. 8 and Supplementary Fig. 9b and c are provided in the Source Data file. Source data are provided with this paper.

## Source data

Source Data
