# Supplementary material for: From radial to unidirectional water pumping in zeta-potential modulated Nafion nanostructures
Source: Nat Commun. 2022 May 19;13:2812. doi: 10.1038/s41467-022-30554-7 (PMC9120507; doi:10.1038/s41467-022-30554-7)
Supplement: Supplementary file 1 — Supplementary Information [file 41467_2022_30554_MOESM1_ESM.pdf]

Supplementary Information

From radial to unidirectional water pumping in zeta-potential modulated Nafion nanostructures

María J. Esplandiu et al.

1. Supplementary Methods

Details of the numerical simulations. We have performed finite element simulations to get more insights on the mechanism controlling the ion-exchange micropumps. The simulations were done using the software COMSOL Multiphysics v4.3. The studies were performed by solving the stationary coupled governing equations:

$$-\varepsilon\nabla^2\varphi = \rho_e \qquad \text{(S.1)}$$

$$\nabla\cdot\boldsymbol{v} = 0 \qquad \text{(S.2)}$$

$$\eta\nabla^2\,\boldsymbol{v} = \nabla p + \rho_e\nabla\varphi \qquad \text{(S.3)}$$

$$\boldsymbol{v}\cdot\nabla C_i = \nabla\cdot(D_i\nabla C_i + z_iF\mu_i\nabla\varphi C_i). \qquad \text{(S.4)}$$

Eq. S.1 is Poisson's equation for electrostatics, relating the local charge density $\rho_e = \sum_i Fz_iC_i$ with the electrostatic potential φ, where ε ($=\varepsilon_r\varepsilon_0$) represents the permittivity of the liquid, F is the Faraday's constant and C_i and z_i are the molar concentration and the valence of each ionic species, respectively, denoted by the subscript i. Eqs. S.2 and S.3 are the stationary Stokes' equations describing the motion of an incompressible fluid at low Reynolds numbers, where $\boldsymbol{v}$, p and η are the fluid velocity, pressure and viscosity, respectively. Finally, Eq. 4 is the stationary Nernst-Planck's equation for mass transport, where D_i is the diffusion coefficient, and μ_i the mobility of the different species. The mobility is connected to the diffusion coefficient through Einstein´s relation, $D_i = \mu_i RT$, where R is the ideal gas constant and T is the absolute temperature.

To avoid charge accumulation and to maintain steady-state conditions, we imposed in the simulations a local current exchange constraint at the Nafion interface, $j_{H^+} = -j_{\mathrm{M}^+}$, i.e., the rate of protons released by the Nafion into the solution must be the same as the rate of uptaken salt cation (M^+). This requirement is implemented in COMSOL simulations through the boundary conditions at the Nafion surface.

In the simulations, four different charged species were considered: protons, hydroxide ions, and the monovalent LiCl salt. The concentration of LiCl used in the simulations was C_d=1.0×10^{-1}mol/m^3.

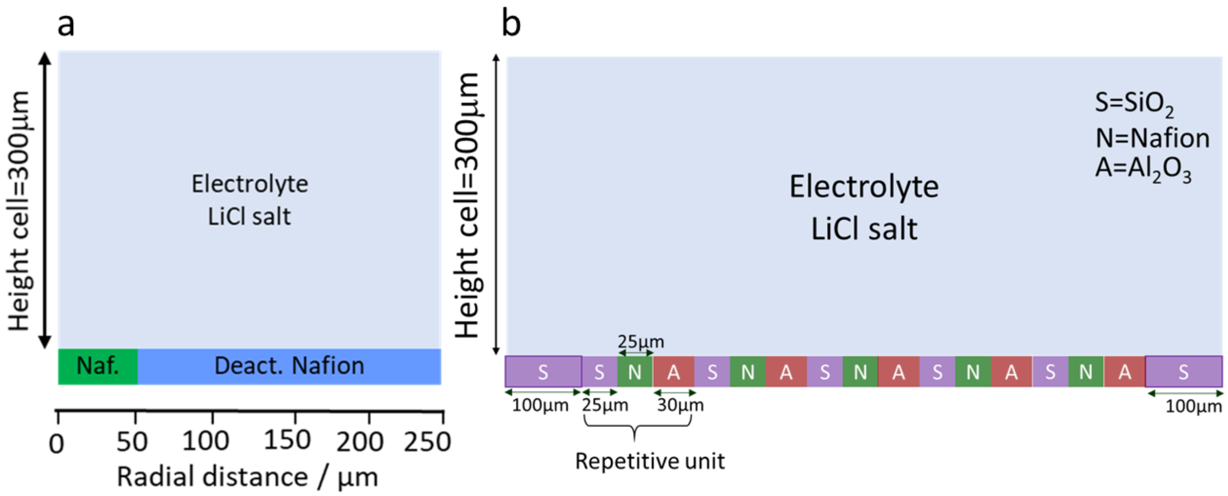

Supplementary Figure 1. Scheme of the layout of the micropump systems used in the simulations. (a) Radial Nafion pump. (b) Micropump array of SiO_2/Nafion/Al_2O_3 strips designed to achieve unidirectional pumping.

The following boundary conditions were defined: $\varphi(z=h)=0$ and $\varphi(z=0)=\zeta_m$, where $h = 300$ µm is the height of the experimental cell and ζ_m represents the zeta potential of the different surfaces. For the fluid velocity, stick boundary conditions were imposed on the substrate and all cell boundaries. For the concentrations of the different species, the bulk values were imposed at the

upper boundary h. The proper boundary conditions to be set for ion exchange at the Nafion interface are not well known. We performed simulations using two different sets of boundary conditions: by assuming that Nafion is a perfect drain for the salt cation[1], i.e., $c_{M^+}(z = 0) = 0$; or treating the ion exchange as a first order reaction characterized by an exchange rate $j_{M^+}(z = 0) = -k_{ex}c_{M^+}(z = 0)$, where k_{ex} is the exchange rate constant[2]. The results obtained with the two types of boundary conditions are similar and become the same in the limit of very large k_{ex}.

Two different micropump geometries, shown in Supplementary Figure 1, have been simulated. The first one, shown in Supplementary Figure 1a, resembles the radial Nafion micropump and consist of a 50 µm Nafion radius disc surrounded by a ring of deactivated Nafion of 200 µm width. Taking advantage of the radial symmetry of this configuration, a 2D axisymmetric domain was implemented. The simulation domain was discretized using an extremely fine mesh with 155250 triangular elements that was refined using 100 boundary layers near the surface. The values used for the zeta potential of the Nafion (ζ_{Nafion} = -73 mV), and deactivated Nafion (ζ_{DN} = -37 mV) regions were the same as the ones measured experimentally by streaming potential/current.

The second simulated system resembles the repeating structure of alternating SiO_2/Nafion/Al_2O_3 strips designed to achieve unidirectional pumping. The geometrical layout of the simulation is illustrated in Supplementary Figure 1b. Given the symmetry of the problem, a 2D section of the real system was implemented as simulation domain, which was discretized using an extremely fine mesh with 903462 triangular elements that was refined using also 100 boundary layers near the surface. Five repeating structures of the basic SiO_2/Nafion/Al_2O_3 pumping unit were simulated flanked by 100 µm of SiO_2 at the beginning and end of the structure. The values used for the zeta

potential of the Nafion (ζ_{Nafion} = -73 mV), SiO_2 (ζ_{SiO2} = -66 mV), and Al_2O_3 (ζ_{Al2O3} = +17 mV) strips were those measured experimentally by streaming potential/current.

A mesh density convergence study was performed to guarantee that discretization errors in the numerical solution were below 5% in all cases. Only stationary solutions were evaluated corresponding to steady state conditions.

Supplementary Table 1 collects the parameters used in the simulations, as well as the dimensions of the 2D models detailed in the work.

Parameter	Description	Value
D_{H^+}	Diffusion coefficient of H^+	$9.3\times10^{-9} m^2/s$
D_{OH^-}	Diffusion coefficient of OH^-	$5.3\times10^{-9} m^2/s$
D_{Li^+}	Diffusion coefficient of Li^+	$1.03\times10^{-9} m^2/s$
D_{Na^+}	Diffusion coefficient of Na^+	$1.33\times10^{-9} m^2/s$
D_{K^+}	Diffusion coefficient of K^+	$1.96\times10^{-9} m^2/s$
$D_{Cd^{2+}}$	Diffusion coefficient of Cd^{2+}	$0.717\times10^{-9} m^2/s$
D_{Cl^-}	Diffusion coefficient of Cl^-	$2.03\times10^{-9} m^2/s$
T	Temperature	298K
R_{Naf}	Radius of Nafion disc	50 µm
w_{Naf}	Width of Nafion strips	25 µm

w_{Al2O3}	Width of Al_2O_3 strips	30 µm
w_{SiO2}	Width of SiO_2 strips	25 µm
C_d	Bulk salt concentration	1.0×10^{-1} mol/m^3
C_b	Water pH (bulk)	5.7
ζ_{Nafion}	Zeta potential Nafion	-73 mV
ζ_{DN}	Zeta potential of deactivated Nafion	-37 mV
ζ_{Al2O3}	Zeta potential Al_2O_3	17 mV
ζ_{SiO2}	Zeta potential SiO_2	-66 mV
H	Cell height	300 µm
ε_r	Relative Permittivity	78
P	Density	10^3kg/m^3
η	Dynamic Viscosity	10^{-3}Pa·s

Supplementary Table 1. Reference parameters used in the simulations

2. Supplementary Figures

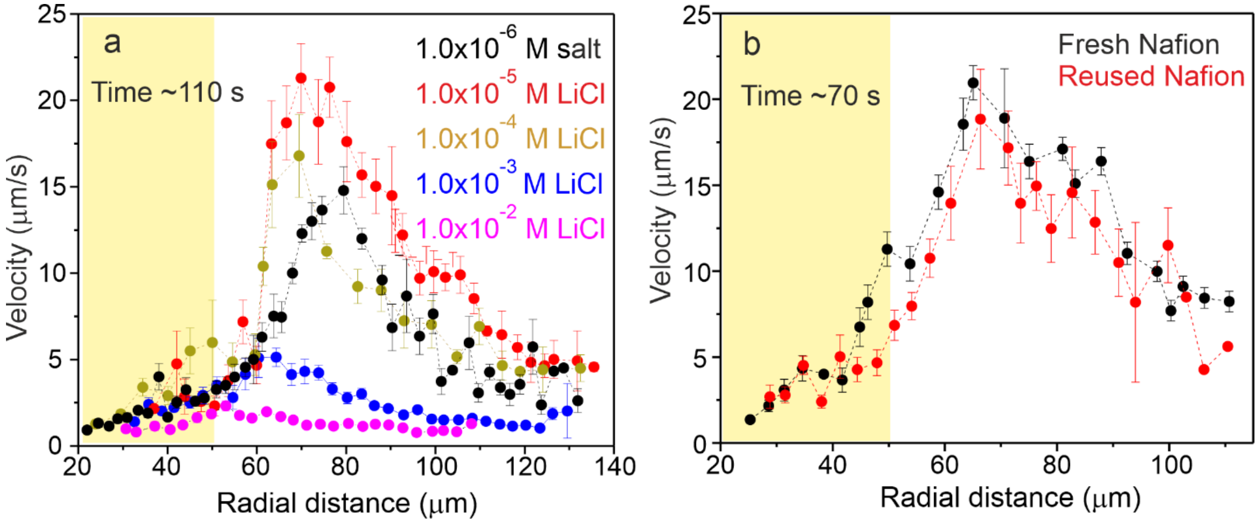

Supplementary Figure 2. Pumping performance as function of salt concentration and regeneration. (a) Radial velocity of polystyrene tracer particles as a function of salt concentration. (b) Comparison between the radial velocity as a function of the radial distance obtained with a fresh Nafion pump (black line and symbols) and with a reused pump (red line and symbols) regenerated after immersion in 1.0×10^{-2}M HCl for 6 hours. Ultrasound either before or during the regeneration process was also used to remove any adsorbed charged tracers of previous experiments. The velocities are very similar, verifying pump reusability. Error bars in the figures represent the standard deviation. Source data are provided as a Source Data file.

Simulations for a radial Nafion pump. Supplementary Figure 3 summarizes the results for the proton concentration, the electric field, and the fluid flow streamlines obtained in the simulation of the radial Nafion pump considering Nafion as a perfect sink for the Li^+ ions. The simulations show that the exchange of protons by Li^+ ions taking place at the Nafion disc generates a concentration gradient and an electric field pointing radially towards the Nafion disc. The

tangential component of this electric field acting on the positive mobile counterions accumulated on the negatively-charged deactivated Nafion surface, drags the fluid towards the Nafion disc generating convection rolls.

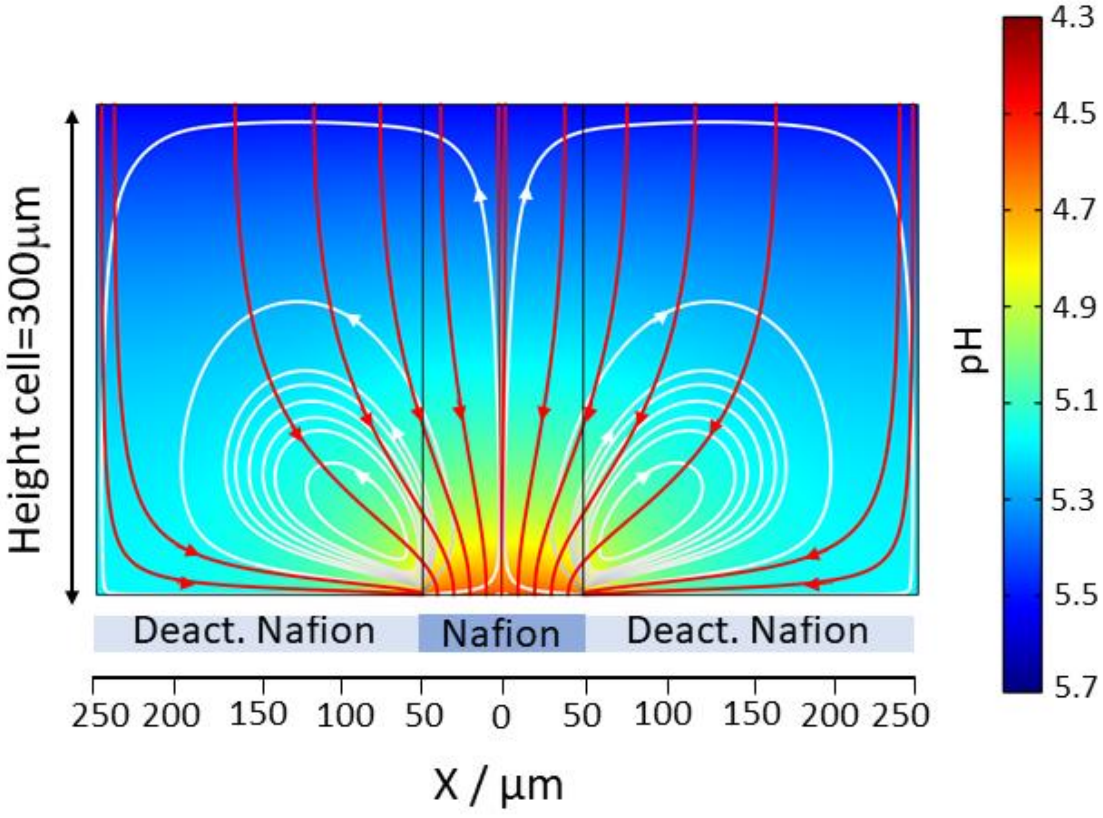

Supplementary Figure 3. Cross-section of the radial Nafion pump showing the values of the proton concentration (color scale), electric field lines (in red) and fluid streamlines (in white) obtained in the simulation.

Supplementary Figure 4 shows the value of the radial component of the electric field as a function of the radial distance, measured from the center of the Nafion disc, obtained at 1 μm above the surface. The radial component of the electric field is negative (i.e. pointing towards the disc center) and shows a strong peak at the disc edge. It is important to clarify that although there

is a zeta potential gradient at the boundary between Nafion and deactivated Nafion, this gradient is not responsible for generating the electric field that moves the fluid. The key parameter in switching on the fluid pumping is the tangential component of the electric field generated by the exchange of ions with different diffusion coefficients. Simulations of Supplementary Figure 4 show that this tangential component of the electric field is almost insensitive to the value of the zeta potential of the material surrounding the active Nafion as can be seen by comparing the simulations in which the zeta potentials of Nafion and deactivated Nafion were used with the ones in which a uniform value of the zeta potential of ζ_{Nafion} = -73 mV was used. The electric field for both cases was basically the same. Moreover, simulations made suppressing the ion-exchange (i.e. setting $k_{ex} = 0$) show that the radial electric field is practically zero and accordingly there is no fluid pumping.

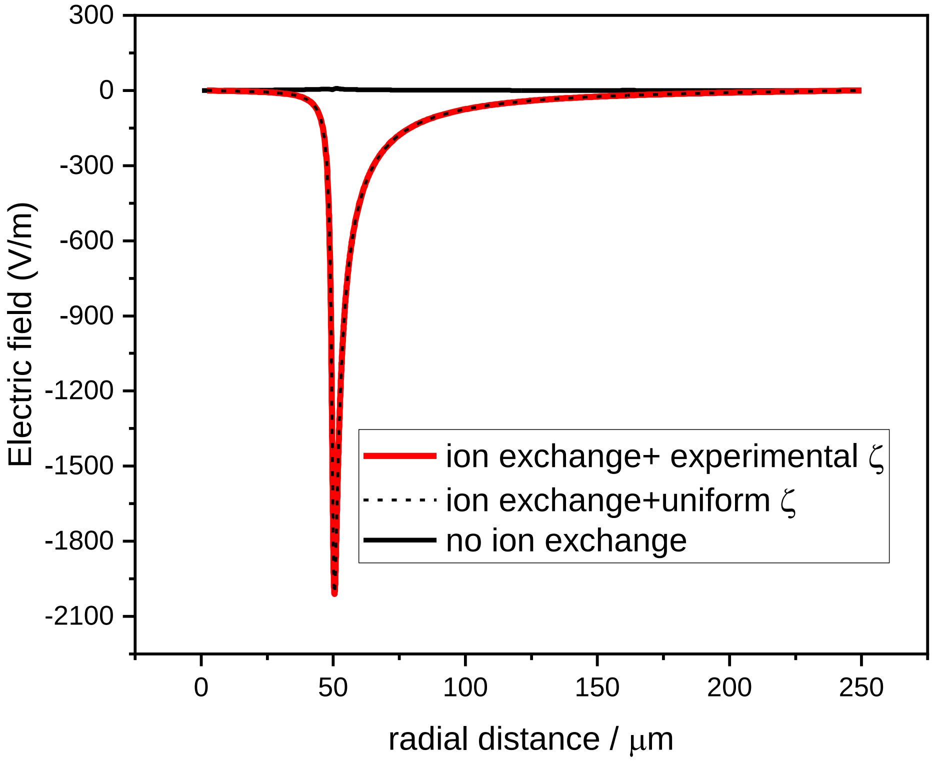

Supplementary Figure 4. Radial component of the electric field as a function of the radial distance for a pump made of a Nafion disc surrounded by deactivated Nafion under different conditions: i) with ion exchange and using the experimental zeta potentials of Nafion and deactivated Nafion; ii) with ion exchange but using a uniform zeta potential (ζ_{Nafion}) on the whole surface; iii) without ion-exchange and using the experimental zeta potentials of Nafion and deactivated Nafion. Source data are provided as a Source Data file.

Supplementary Figure 5 shows the effect of the salt concentration of LiCl on the electric field and fluid flow. The electric field and fluid flow increase with salt concentration up to reach saturation at concentrations above $1.0x10^{-4}$ M. Remarkably, the fluid velocity is predicted to persist even at 100 mM of salt concentration.

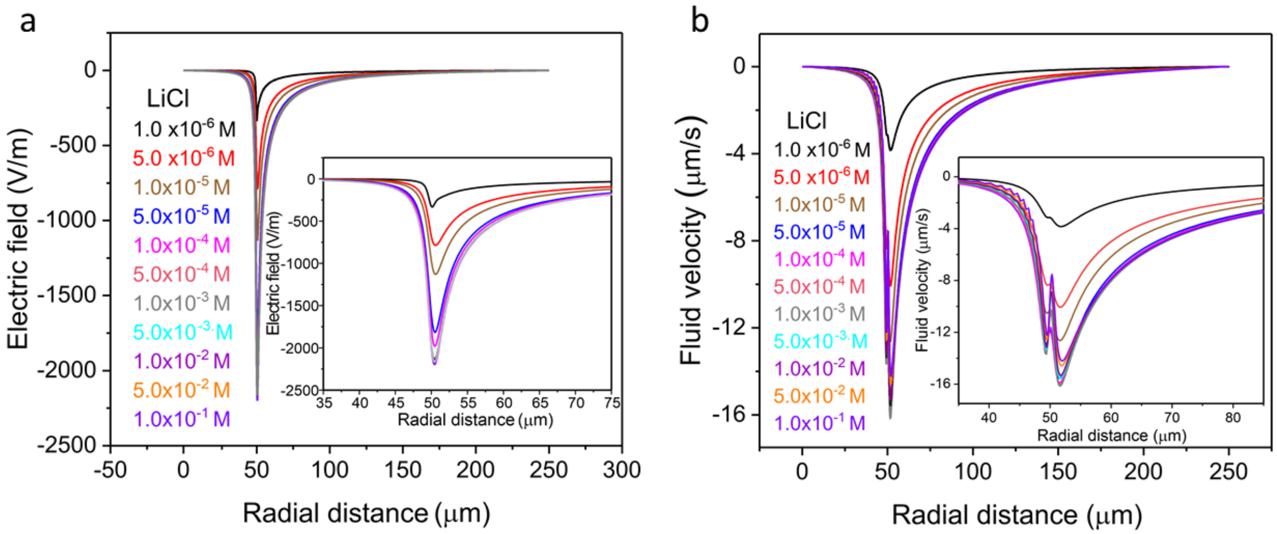

Supplementary Figure 5. Radial component of the electric field (a) and fluid flow (b) at 1 μm above the surface as a function of the radial distance for a pump made of a Nafion disc surrounded by deactivated Nafion in presence of different concentrations of LiCl. The inset shows a zoomed region of the plot around the disk edge. The simulations were performed considering Nafion as a perfect drain for the salt cation. Source data are provided as a Source Data file.

The effect of different salts of alkali metal chlorides and $CdCl_2$ on the electric field and fluid flow is simulated in Supplementary Figure 6. It can be observed that the electric field and consequently the fluid velocity increase as the diffusion coefficient of the salt cation decreases (D_{K+1} = 1.96x10^{-9}m^2/s, D_{Na+} =1.33x10^{-9}m^2/s, D_{Li+1} =1.03x10^{-9}m^2/s, D_{Cd2+} =0.717x10^{-9}m^2/s). The simulations also support the experimental finding that the Nafion pumps can be triggered by different salts with similar performances.

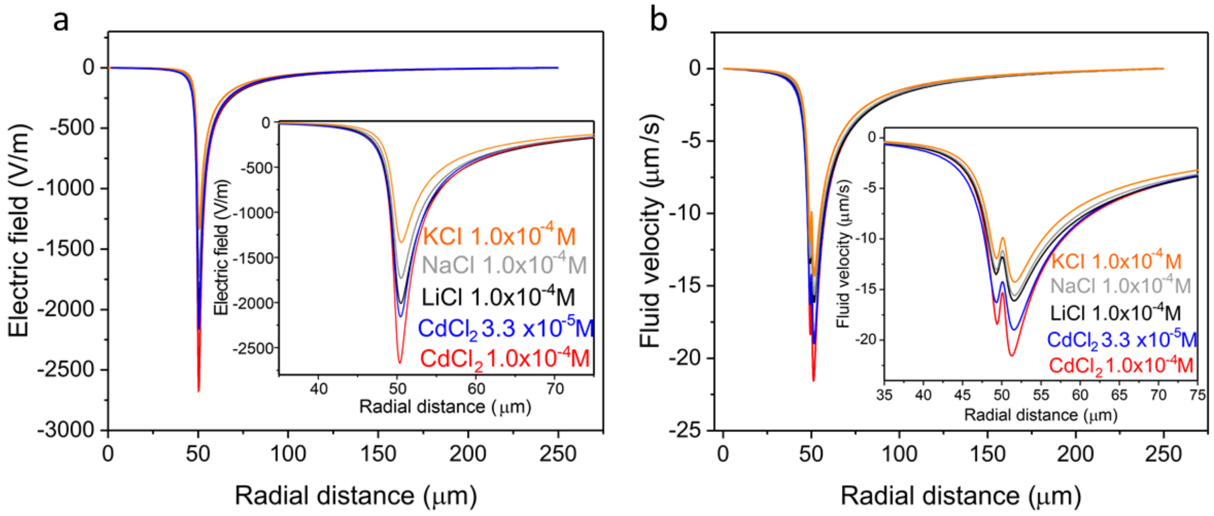

Supplementary Figure 6. Radial component of the electric field (a) and fluid flow (b) at 1μm above the surface as a function of the radial distance for a pump made of a Nafion disc surrounded by deactivated Nafion in presence of different monovalent and divalent ($CdCl_2$) salts of the same concentration 1.0x10^{-4}M and with $CdCl_2$ at a concentration of 3.3x10^{-5}M, corresponding to the same ionic strength than the monovalent salts. The inset shows a zoomed region of the plot around the disk edge. The simulations were performed considering Nafion as a perfect drain for the salt cation. Source data are provided as a Source Data file.

Simulations of the SiO_2/Nafion/Al_2O_3 micropump array. Supplementary Figure 7 shows the value of the tangential component of the electric field along the micropump strip array measured at 3 μm above the surface. The origin of the X coordinate has been placed at the start of the first SiO_2/Nafion/Al_2O_3 repeating unit. The tangential electric field is positive at the SiO_2/Nafion boundary, pointing towards Nafion. The tangential electric field is negative at the Nafion/Al_2O_3 border indicating that is also pointing towards Nafion and therefore promoting the fluid flow from Nafion towards the region of positive zeta potential provided by Al_2O_3.

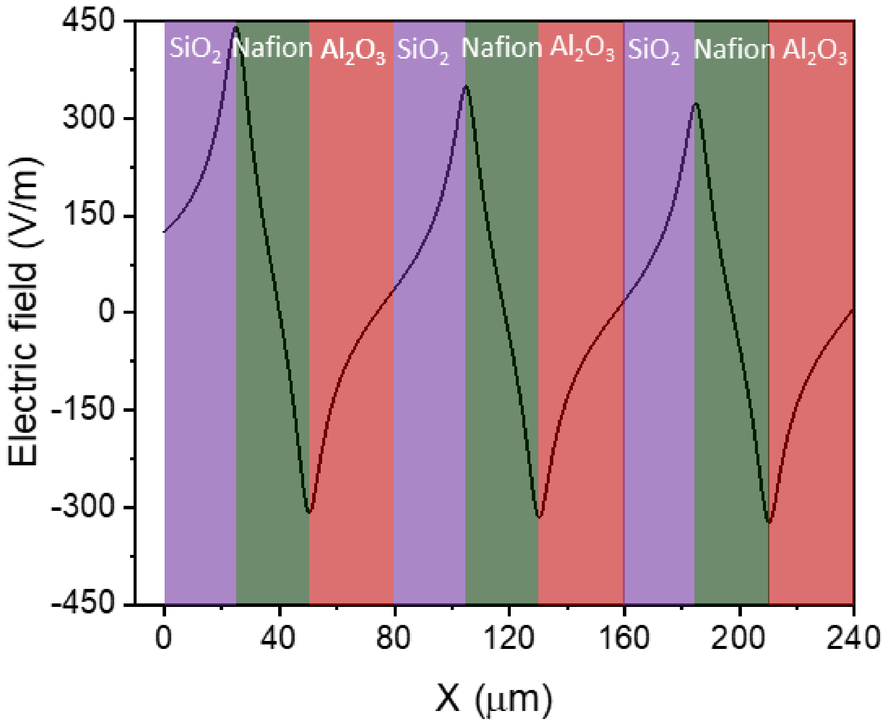

Supplementary Figure 7. Simulated electric field profile along the micropump array at a z height of 3 μm and for a constant ion exchange rate of $k_{ex} = 10^{-5}\ m/s$. Source data are provided as a Source Data file.

Pump performance with thicker Nafion structures. A radial Nafion pump with a thicker Nafion layer (2 μm) was fabricated and its performance was evaluated in a solution of 1.0×10^{-4} M LiCl. Supplementary Figure 8 shows that for this thicker Nafion device, the operation of the pump extends far beyond the 45 minutes achieved with a 600 nm thick Nafion pump. The velocity decay is much slower in the case of the pump with a thicker Nafion layer, becoming operational for more than two hours. These results corroborate that thicker Nafion layers delay the saturation of the pump and can sustain the pumping at high velocities for longer times.

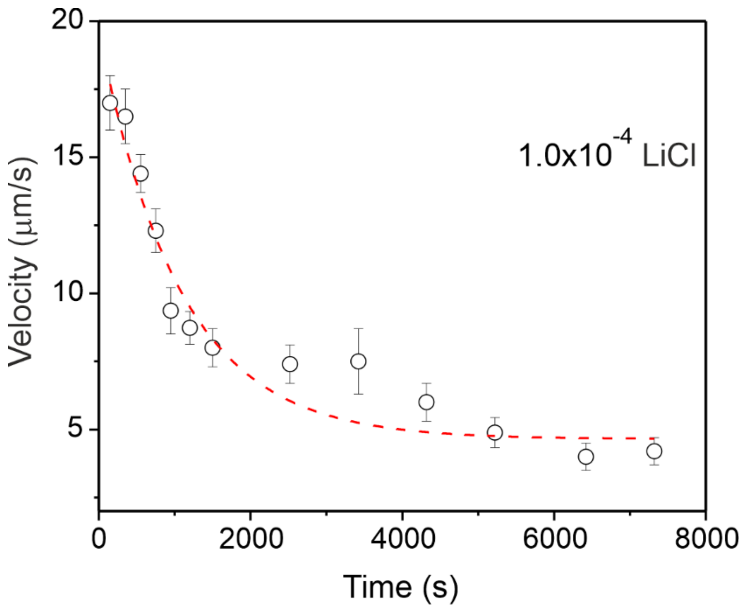

Supplementary Figure 8. Time evolution of the maximum radial velocity of tracers averaged over 25-50 trajectories in presence of 1.0x10^{-4} M LiCl for a 500 µm Nafion disc of 2µm thick deposited on a previous patterned gold disc produced by stencil lithography on a silicon wafer. Error bars in the figure represent the standard deviation. Source data are provided as a Source Data file.

Unidirectional pumping with other arrangement of strip arrays. A cross-shaped strip array configuration has been designed to test that fluid can be redirected simultaneously in different directions. Supplementary Figure 9 a shows a picture of part of the cross with the patterned structures and also a scheme of the repeating unit of the bands (SiO_2/Nafion/Al_2O_3). Supplementary Figures 9 b and c show the trajectories of different tracers observed in the longitudinal (a) and vertical (b) arms of the cross-shaped structure. Clearly, the fluid flows in nearly orthogonal directions at the different strip orientations driven by the underlaid patterned micropumping structure. The trajectories of the image c) are affected by a small horizontal drift

generated by longitudinal arm of the cross, which generates large horizontal fluid flows, specially at the initial stages of the experiment.

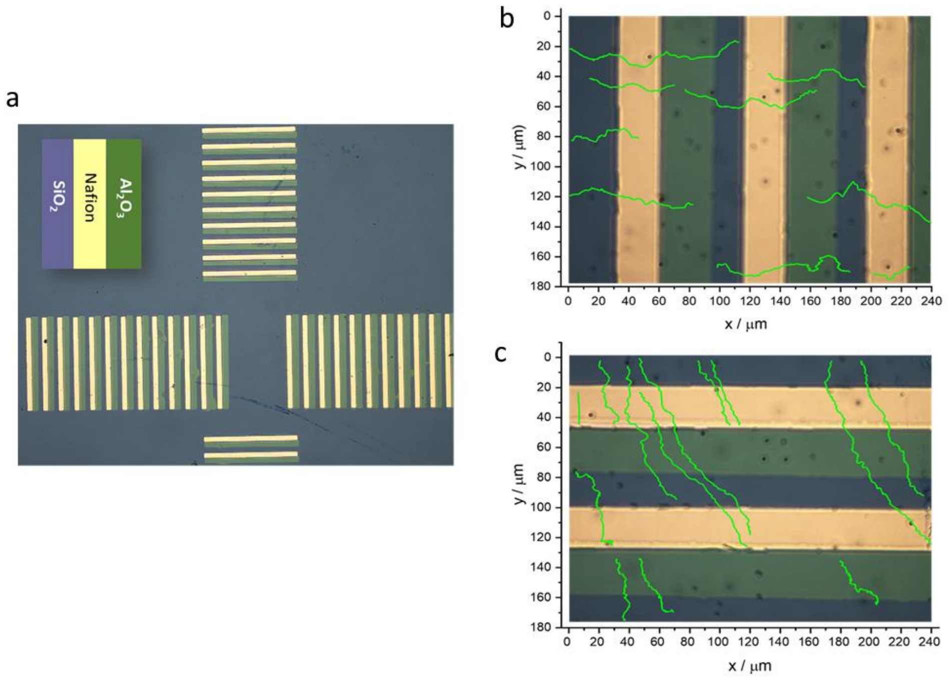

Supplementary Figure 9. a) Patterned array of strips forming a cross made from the repetitive unit composed of SiO_2/Nafion /Al_2O_3 as indicated in the insert. b) and c) Tracer trajectories along the patterned surface at the longitudinal (b) and vertical (c) arms of the cross, where the strip array is arranged vertically and horizontally, respectively. Source data of (b) and (c) are provided as a Source Data file.
