# Supplementary material for: From radial to unidirectional water pumping in zeta-potential modulated Nafion nanostructures
Source: Nat Commun. 2022 May 19;13:2812. doi: 10.1038/s41467-022-30554-7 (PMC9120507; doi:10.1038/s41467-022-30554-7)
Supplement: Supplementary file 2 — Description of Additional Supplementary files [file 41467_2022_30554_MOESM2_ESM.docx]

Description of Additional Supplementary Files

File Name: Supplementary Movie 1

Description: Motion of polystyrene tracers in 1.0x10^-4^M LiCl. The movie was taken at Z distances close to the surface of the micropump system. A radial fluid flow drags the particles inwards the Nafion disc which has not so much contrast to optically visualize it in presence of the liquid.

File Name: Supplementary Movie 2

Description: Motion of polystyrene tracers in 1.0x10^-4^M LiCl, taken at high Z distances from the surface (Z=100 µm) of the micropump system. Now the radial fluid flow lifts up the particles at the Nafion disc and drags them outwards the Nafion disc.

File Name: Supplementary Movie 3

Description: Motion of polystyrene tracers in 1.0x10^-4^M NaCl taken at close distances from the surface of the micropump system.

File Name: Supplementary Movie 4

Description: Motion of polystyrene tracers in 1.0x10^-4^M CdCl_2_ taken at close distances from the surface of the micropump system.

File Name: Supplementary Movie 5

Description: Motion of polystyrene tracer particles in 1.0x10^-4^M LiCl at the micropump array composed of alternating strips of deactivated Nafion/Nafion/Al_2_O_3_. The movie has been speeded up (x 4).

File Name: Supplementary Movie 6

Description: Motion of polystyrene tracer particles at the micropump array composed of alternating strips of SiO_2_/Nafion/Al_2_O_3_ in 1.0x10^-4^M LiCl. The movie has been speeded up (x4).

File Name: Supplementary Movie 7

Description: Motion of polystyrene tracer particles at the micropump array composed of alternating strips of SiO_2_/Nafion/Al_2_O_3_ but in absence of salts. The tracer particles were purified by dialysis to remove any salt residues from their synthesis.

File Name: Supplementary Movie 8

Description: Motion of polystyrene tracer particles at the micropump array composed of alternating strips of SiO_2_/Nafion/Al_2_O_3_ in a cross configuration in presence of 1.0x10^-4^M LiCl. The movie has been speeded up (x4).
